# Comparison of the effects of propofol and alfaxalone on the electrocardiogram of dogs, with particular reference to QT interval

**DOI:** 10.3389/fvets.2023.1330111

**Published:** 2024-01-08

**Authors:** Vincenzo Casoria, Victoria Greet, Adam Auckburally, Steve Murphy, Derek Flaherty

**Affiliations:** ^1^Department of Anaesthesia and Analgesia, Southern Counties Veterinary Specialists, Ringwood, United Kingdom; ^2^Department of Cardiology, Southern Counties Veterinary Specialists, Ringwood, United Kingdom

**Keywords:** anesthesia, propofol, alfaxalone, electrocardiogram, arrhythmias, QT interval

## Abstract

Cardiac electrical activity is often altered by administration of anesthetic drugs. While the effects of propofol in this regard have previously been described in dogs, to date, there are no reports of the effect of alfaxalone. This study investigated the impact of both propofol and alfaxalone on the ECG of 60 dogs, after premedication with acepromazine and methadone. Heart rate increased significantly in both groups. The PR and QRS intervals were significantly increased following propofol while with alfaxalone the QRS duration was significantly increased and ST segment depression was observed. The QT and JT interval were significantly shorter following induction with alfaxalone, but, when corrected (c) for heart rate, QTc and JTc in both groups were significantly greater following induction. When comparing the magnitude of change between groups, the change in RR interval was greater in the alfaxalone group. The change in both QT and JT intervals were significantly greater following alfaxalone, but when QTc and JTc intervals were compared, there were no significant differences between the two drugs. The similarly increased QTc produced by both drugs may suggest comparable proarrhythmic effects.

## Introduction

Continuous electrocardiographic (ECG) monitoring is essential in the anesthetized patient for diagnosis of peri-operative arrhythmias. These arrhythmias may be secondary to spontaneous or drug-induced changes in the cardiac electrical activity (1). While the incidence of perioperative arrhythmias in people is extremely high (2, 3), only limited comparable information is available in dogs, with one study reporting a prevalence of 2.5% during general anesthesia (4). In particular, changes in the QT interval are associated with potential arrhythmogenesis. The QT interval is measured from the onset of the QRS to the end of the T wave, and represents the time taken for ventricular depolarization (QRS) and repolarization (JT interval). The JT interval is the time between the J point, where the QRS complex joins the ST segment, and the end of the T wave. Typically, prolongation of the QT interval reflects an increase in the duration and heterogeneity of ventricular repolarization (5). This may lead to the development of potentially malignant arrhythmias through triggered activity, re-entry or both (6). Torsades de pointes are a well-reported consequence of QT prolongation in both dogs and humans (7, 8), and may lead to sudden death. QT prolongation may be congenital or drug-induced. Although congenital QT prolongation has been described in dogs (9), it is less frequently described compared to humans, where the incidence is ~1 in 2,000 (10). A large number of drugs potentially prolong the QT interval (11), and the impact of a new drug on ventricular repolarization still remains a critical step in the overall development process (12). Propofol and alfaxalone are the most popular intravenous anesthetics used in small animal anesthesia, and the former is the most widely used human anesthetic induction agent. Although relatively few studies have examined the effects of propofol on the ECG in dogs, it has been described as potentially arrhythmogenic in this species (13, 14) while, in humans, it seems to display both pro- and antiarrhythmic effects in a concentration-dependent manner (15). In both species, the QT interval is prolonged (14, 16) while no increase in QT dispersion has been found after propofol administration in dogs (14). Alfaxalone in conjunction with alfadolone has been previously approved for use in humans and cats. However, it was removed from the human market, and later the veterinary market due to a high incidence of anaphylactic reactions to its excipient, Cremophor EL (17). Alfaxalone was reformulated in 2-hydroxypropyl-b-cyclodextrin, a synthetic carbohydrate molecule not associated with allergic reactions, and is licensed for use in dogs and cats. The pharmacological and anesthetic properties of alfaxalone in dogs have been reviewed (18), but no studies have evaluated the effects of alfaxalone on the ECG parameters in dogs, and very few studies have assessed the effects on the ECG in humans (19, 20). A reformulated version of alfaxalone also using a cyclodextrin excipient, is currently undergoing human trials for re-introduction as an anesthetic agent in this species, so it would be interesting to assess the effects of alfaxalone on the canine ECG with particular reference to the QT interval as a guide to possible effects in humans. The aim of this study was to compare the effects of propofol and alfaxalone on ECG parameters in healthy dogs undergoing general anesthesia, with emphasis on the QT interval.

## Materials and methods

This study received ethical approval from the RCVS Ethics Review Panel (Ref: 2021-34), and an Animal Test Certificate was obtained from the Veterinary Medicines Directorate (ATC-S-164). Informed client consent was obtained for all animals recruited to the study.

Sixty healthy adult dogs (American Society of Anesthesiologists status I or II) were included. Aggressive animals, those sedated or anesthetized during the previous 24 h, those with a recent history of vomiting or regurgitation, or dogs with known structural or functional cardiac disease or pre-existing arrhythmias, were excluded. Brachycephalic dogs and dogs which were deemed to be significantly underweight (BCS <4/9) or overweight (BCS >6/9) were also excluded.

Each dog received a complete physical examination and were randomly assigned, using computer-generated random numbers, to group A for anesthetic induction with alfaxalone (10 mg/ml Alfaxan; Jurox, UK), or group P for induction with propofol (10 mg/ml Propoflo Plus; Zoetis, USA). Premedication consisted of 0.03 mg/kg acepromazine (2 mg/ml Acesedate; Jurox, UK) with 0.3 mg/kg methadone (10 mg/ml Comfortan; Dechra, UK) administered intramuscularly in the cervical epaxial musculature. Following this, the dog was moved to a quiet environment for 30 min prior to aseptic intravenous (IV) cannula placement in an appropriate limb. The dog was then gently restrained in right lateral recumbency and allowed a short period of acclimatization.

Non-traumatic electrodes were used to obtain a complete 12 lead ECG. Six limb leads (I, II, III, aVR, aVL and aVF) were used. The precordial lead system (V1, V2, V3, V4, V5, V6) was utilized, with lead V1 positioned at the costochondral junction of the right, first intercostal space (21). The six limb leads were recorded simultaneously with the six chest leads for 30 s at a paper speed of 50 mm/s and voltage of 20 mm/mV.

Following acquisition of the ECG, the dog was preoxygenated with 100% oxygen via face mask for at least 3 min and then induced with either alfaxalone or propofol depending on group assignation, administered IV by hand, to effect. The same anesthetist (VC) performed all inductions with an end-point of loss of consciousness, absent palpebral reflex and loss of jaw tone. A further ECG trace was then taken for 30 s at the same paper speed and gain, with continued administration of 100% oxygen via facemask throughout the recording. Once the ECG recording was completed, the trachea was intubated with an appropriately sized endotracheal tube and anesthesia continued for the planned procedure.

All the ECG measurements were made according to a standardized approach (22, 23). The QT interval was measured from the earliest QRS onset in any lead of the six simultaneously recorded leads to the intersection between the maximal downslope of the terminal portion of the T-wave to the isoelectric baseline (24, 25). All the measurements of PR interval, QRS interval, ST segment deviation from baseline, T-wave amplitude and QT interval were taken from lead II, and recorded in each case as the mean values from three consecutive complexes. The JT interval was calculated as the QT interval minus the QRS interval. Rate-corrected QT and JT intervals (QTc and JTc) were obtained by using the formula *QTc* = *QT* − 0.087 (*RR* – 1, 000) (26) and *JTc* = *QTc* − *QRS*. The RR interval used in this calculation was taken as the mean RR interval across the complexes recorded over 6–10 s (9).

QT dispersion (QTd) was calculated as the difference between the maximum and minimum QT interval observed across all the 12 leads of the ECG (27). All the measurements were made by the same observer (VC).

### Statistical analysis

A power analysis was performed based on a recent study (28) comparing the effects of methadone or hydromorphone on cardiac conductivity, and which used a clinically significant difference of 20 ms and demonstrated a pooled variance of 25 ms on QT interval. Using this data, and based on a power of 80% and alpha of 0.05, 52 dogs were required for our study. Given the potential for some ECG traces to be unsuitable for final analysis (e.g., due to movement artifact), we elected to recruit 60 dogs in total, 30 in each group. Data were analyzed in Excel spreadsheet format (Microsoft Excel for Mac, version 16.16.27) using MedCalc (version 17.1 64-bit, Ostend, Belgium). All data were tested for normality using the Shapiro Wilk test and diagnostic plots. Age and bodyweight between groups were tested using ANOVA. Paired data for ECG variables before and after induction to general anesthesia were tested using Student's *t*-test. The magnitude of changes was calculated and compared between the two groups using independent *t*-tests. A *p*-value of <0.05 was considered statistically significant.

## Results

In total, 120 ECG traces were collected with 12 traces excluded due to the presence of artifacts, and 108 ECG were included for statistical analysis (27 dogs in each group). All data were normally distributed. No arrhythmias were detected in the propofol group. One dog in the alfaxalone group demonstrated isolated ventricular escape complexes after premedication, that were not present after induction.

Patient variables, breed distribution and dose of propofol and alfaxalone are described in Table 1. There were no statistically significant differences detected for age and body weight between groups.

**Table 1:** Animal variables in 54 dogs, premedicated using acepromazine (0.03 mg/kg) and methadone (0.3 mg/kg) intramuscularly and induced to general anesthesia with alfaxalone or propofol.

	**Alfaxalone group (*n* = 27)**	**Propofol group (*n* = 27)**
Age (months)	78.2 (± 44.4)	68.7 (± 36.2)
Weight (kg)	20.3 (± 9.8)	21.0 (±10.8)
Breed distribution	Pomeranian (1) Springer spaniel (2) Golden retriever (1) Labrador retriever (5) Cross breed (3) Dachshund (2) Cocker spaniel (4) Portuguese water dog (1) Labradoodle (1) German shepherd (1) Poodle (2) Cavachon (1) Cockapoo (2) Italian spinone (1)	Cross breed (5) Cocker spaniel (2) Dalmatian (1) Golden retriever (2) Staffordshire bull terrier (1) Jack Russell terrier (2) Cavalier King Charles spaniel (1) Labrador retriever (3) Shetland sheepdog (1) Border collie (1) Sprocker (1) Dachshund (1) Bichon frise (1) Springer spaniel (1) Irish setter (1) Pomeranian (1) Spanish Greyhound (1) Leonberger (1)
Dose (mg/kg)	1.9 (± 0.5)	2.4 (±0.7)

Age, weight and anesthetic drug dose are presented as mean ± SD.

Measurements from the ECG analysis are reported in Table 2. Heart rate was similar between groups following premedication, but increased significantly in both groups following induction. An increase in the QRS duration was documented in both alfaxalone and propofol groups following induction, although this did not exceed the upper limits of the reported reference interval. A significant increase in PR interval duration was only documented in the propofol group post-induction. A significant ST segment deviation was only observed after alfaxalone administration.

**Table 2:** Electrocardiogram variables (mean ± SD) in 54 dogs, 30 min after premedication using acepromazine (0.03 mg/kg) and methadone (0.3 mg/kg) intramuscularly and after induction to general anesthesia with alfaxalone or propofol.

**ECG variable**	**Alfaxalone group (*****n*** = **27)**			**Propofol group (*****n*** = **27)**		
	**Pre-induction**	**Post-induction**	***p-*** **value**	**Pre-induction**	**Post-induction**	***p-*** **value**
Heart rate (bpm)	61.5 (±18.1)	94.1 (±29.5)	< 0.0001	69.8 (±18.6)	84.6 (±24.4)	< 0.0001
RR interval (ms)	1048.95 (±272.86)	693.86 (±196.27)	< 0.0001	919.05 (±241.18)	764.23 (±204.53)	< 0.0001
PR interval (ms)	109.36 (±19.42)	109.00 (±18.53)	0.9	98.75 (±15.72)	103.41 (±15.28)	< 0.001
QRS width (ms)	62.99 (±5.5)	64.06 (±6.25)	0.02	61.04 (±5.54)	62.21 (±5.97)	< 0.01
ST segment deviation (mV)	−0.05 (±0.07)	−0.08 (±0.08)	0.04	−0.08 (±0.08)	−0.10 (±0.09)	0.15
T wave amplitude (mV)	−0.06 (±0.35)	0.05 (±0.36)	< 0.01	0.04 (±0.32)	0.04 (±0.41)	0.98
Positive T wave amplitude	0.23 (±0.12)	0.30 (±0.17)	0.08	0.26 (±0.14)	0.26 (±0.23)	0.92
Negative T wave amplitude	−0.36 (±0.23)	−0.22 (±0.32)	0.05	−0.34 (±0.13)	−0.33 (±0.39)	0.94
QT interval (ms)	248.53 (±21.57)	235.37 (±24.78)	< 0.0001	239.93 (±22.17)	240.11 (±22.28)	0.9
QTc interval (ms)	244.27 (±20.85)	262.00 (±15.58)	< 0.0001	246.97 (±17.62)	260.62 (±13.74)	< 0.0001
JT interval (ms)	185.54 (±19.67)	171.31 (±22.59)	< 0.0001	171.07 (±19.77)	177.90 (±20.65)	0.49
JTc interval (ms)	181.28 (±21.56)	197.94 (±14.58)	< 0.0001	185.93 (±15.38)	198.41 (±11.81)	< 0.0001
QT dispersion (ms)	21.94 (±6.15)	22.17 (±7.81)	0.85	21.79 (±10.12)	21.48 (±6.47)	0.86

**Figure 1:**
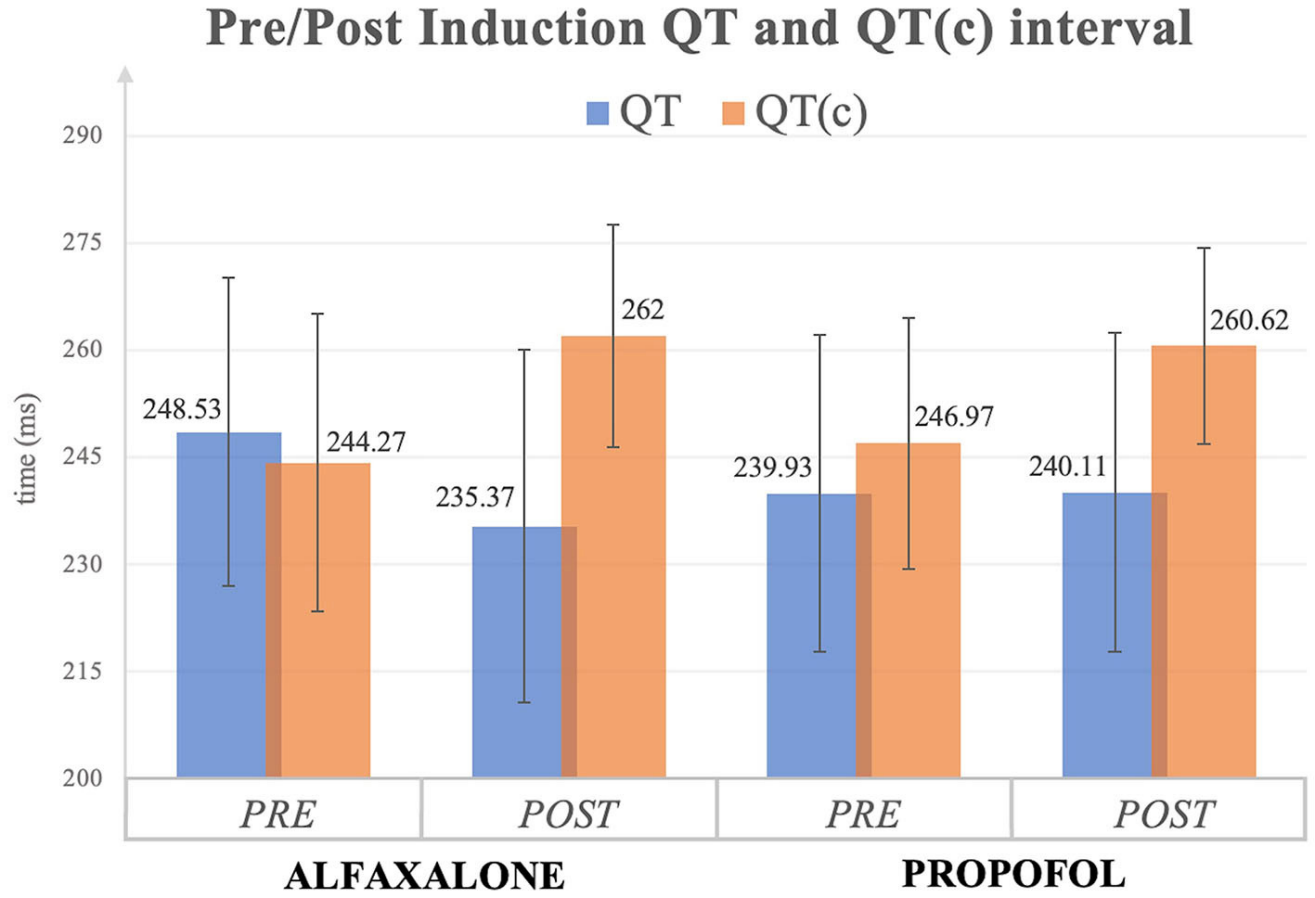
QT and QT corrected (c) interval (mean ± SD) before and after induction to general anesthesia with alfaxalone or propofol. All values are expressed as milliseconds (ms).

In the alfaxalone group, QT interval was significantly shorter following induction, but once corrected for heart rate (QTc), the interval in both groups was significantly greater following induction (Figure 1). Similarly, JT interval decreased in the alfaxalone group, in contrast to JTc which increased in both groups following induction. No significant change to QTd was seen in either group. In one dog in each group, one of the 12 leads was of insufficient quality to determine where the T wave ended, and this lead was excluded when calculating QTd; however, 11 leads were considered sufficient (14).

The T wave amplitude increased significantly in the alfaxalone group; however, due to the variation in polarity of the T wave from animal to animal, the T waves were grouped into positive and negative polarities and then re-tested, following which there were no significant differences in T wave amplitude either within or between the groups.

When comparing the magnitude of change between groups (Table 3), the change in RR interval was greater in the alfaxalone group. The change in both QT and JT intervals were significantly more in the alfaxalone group compared with the propofol group. However, when the corrected QT and JT intervals were compared, there were no significant differences.

**Table 3:** Electrocardiogram variables (mean ± SD) in 54 dogs, 30 min after premedication using acepromazine (0.03 mg/kg) and methadone (0.3 mg/kg) intramuscularly and after induction to general anesthesia with alfaxalone or propofol.

**ECG variable**	**Mean difference alfaxalone group (*n* = 27)**	**Mean difference propofol group (*n* = 27)**	***p*-value**
RR interval (ms)	355.1 (±207.4)	154.8 (±129.0)	0.0001
PR interval (ms)	0.36 (±15.1)	−4.7 (±6.5)	0.12
QRS interval (ms)	−1.1 (±2.2)	−1.2 (±2.1)	0.87
ST segment deviation (mV)	0.02 (±0.06)	0.01 (±0.05)	0.43
T wave amplitude +ve (mV)	−0.07 (±0.15)	0.002 (±0.14)	0.15
T wave amplitude –ve (mV)	−0.14 (±0.23)	−0.008 (±0.37)	0.29
QT interval (ms)	13.2 (±11.9)	−0.19 (±7.8)	< 0.0001
QTc interval (ms)	−17.7 (±17.8)	−13.7 (±9.6)	0.30
Jt interval (ms)	14.2 (±11.8)	0.24 (±7.7)	< 0.0001
Jtc interval (ms)	−16.7 (±17.4)	−13.2 (±11.2)	0.39
QT dispersion (ms)	−0.24 (±6.4)	1.04 (±15.8)	0.70

For each variable, results are reported as mean difference before and after induction, with a positive number indicating a decrease post-induction compared to the pre-induction value.

## Discussion

This study expands previous knowledge of the ECG effects of propofol in dogs, and is the first to investigate those of alfaxalone in this species. Given the potential for the latter to receive licensing authorization for use in humans, our results provide a preliminary basis for assessing the potential proarrhythmic effects of alfaxalone in this species.

In this study, a positive chronotropic effect was observed following administration of both propofol and alfaxalone, with higher heart rates observed with alfaxalone. In healthy dogs, clinical doses of alfaxalone have minimal cardiovascular effects: cardiac output (CO) is usually well maintained (29, 30), while arterial blood pressure (ABP) and systemic vascular resistance (SVR) may decrease (29–32), but usually remain in the physiological range. It has been hypothesized that, due to the preservation of the baroreceptor reflex, alfaxalone administration results in a greater increase of heart rate (HR) compared with propofol. This represents a compensatory mechanism to maintain CO and ABP following the decrease in SVR (30, 31). Drugs used for premedication can influence this response, and depending on the protocol used, HR in dogs was reported to increase (32, 33), decrease (34) or remain unchanged (32, 35, 36) following induction with alfaxalone. While propofol is generally reported in humans to cause a reduction in HR (37), other investigators have demonstrated positive chronotropism (38), albeit not as marked as with other drugs such as alfaxalone and thiopentone as shown in some studies in dogs (14, 29). This was observed in our study and is thought to be a result of the differing effects of the drugs on baroreceptor sensitivity, as propofol is reported to cause a complete resetting of the baroreceptor reflex (39). Preservation of the baroreceptor reflex may explain a marked sympathetic response to vasodilation when alfaxalone is administered compared to propofol.

The PR interval reflects the time taken for conduction through the atrioventricular (AV) node, and tends to increase with decreasing HR and shortens as HR increases (40). Prolongation of the PR interval, known as first-degree atrioventricular block (1AVB), is usually well tolerated, but may be exacerbated by drugs or diseases that alter autonomic tone (23). One dog in the propofol group and four dogs in the alfaxalone group demonstrated 1AVB pre-induction, but normalized in two dogs in the alfaxalone group after induction. One further dog in the alfaxalone group developed mild 1AVB post-induction. It is possible that this may have been caused by the drugs used for premedication. There are no published studies assessing the effects of acepromazine on conduction through the AV node. Full mu opioid agonists are known to result in vagally mediated bradycardia (41). In addition, the chemical structure of methadone resembles that of calcium channel blocking agents (42, 43), resulting in functional L-type calcium channel blockade. These characteristics of methadone create the potential for 1AVB, and this has been shown in dogs (28). Following induction with propofol, the PR interval was significantly prolonged compared to pre-induction values. As 5 dogs demonstrated 1AVB pre-induction, we decided to re-analyse the data with these animals excluded to remove any potential influence of premedication on the induction agent effect. However, induction with propofol still resulted in a significantly prolonged PR interval. This is in contrast to the findings of Dennis et al. (14) where the PR interval was unchanged following propofol administration and the reasons for these dissimilar results are unclear. Previous animal studies have examined the effects of propofol on sinus and AV nodal function with conflicting results (44–46). The majority of studies in human medicine did not find any direct effects of propofol on the activity of the AV conduction (47, 48). In contrast, some electrophysiology studies in children, scheduled for radiofrequency ablation, confirm a statistically significant prolongation of the AV node conduction caused by propofol (49, 50). Further studies may be warranted to investigate this effect in dogs.

The QRS complex reflects depolarization of the ventricular myocardium, and in dogs, a wide QRS is defined as >70 ms (22, 23). An increased QRS complex duration pre-induction was observed for four dogs that was still present post-induction, with one dog having a QRS duration of 70.33 ms following alfaxalone administration. Although there was a statistically significant increase in QRS duration in both alfaxalone and propofol groups, all remaining dogs demonstrated values pre- and post-induction that were within the normal range. A number of pathological conditions may cause QRS prolongation, and although we cannot completely exclude the presence of mild subclinical cardiac disease, it is unlikely in this population of dogs. Methadone and acepromazine do not influence QRS complex duration in studies conducted in dogs (51, 52). Therefore, it is unlikely that the drugs used for premedication in our study were responsible for the QRS prolongation observed before induction. Again, data were reanalyzed when these dogs were removed to account for any influence from premedication and this did not change our results. Evidence of a possible interaction between alfaxalone and cardiac ion channels and electrical conductivity is lacking. Although the effects of propofol on inward/outward ion currents have been described (15, 53, 54), no change in QRS duration was observed in a human study that evaluated the effects of propofol on electrical activity in the heart (55). Furthermore, propofol did not affect the QRS interval in healthy dogs premedicated with acepromazine and pethidine (14), which is in contrast with the findings from our study. The reasons for these differences are not immediately apparent but it is important to reiterate that the QRS prolongation found in our study remained within the normal range reported for dogs.

This study showed a significant decrease of QT and JT intervals in dogs induced with alfaxalone. The duration of the QT and JT interval varies with HR: the faster the heart rate, the shorter the interval (56). Therefore, the more marked chronotropic effect of alfaxalone is the reason why these intervals decreased significantly in this group. Due to the effect of HR on the QT and JT intervals, it is more appropriate to calculate corrected intervals. This is a correction based on the R–R interval or average HR, depending on the formula used. Many different formulae have been developed (6), but there is no accepted gold standard in dogs. In this study, Van de Water's linear formula was used (26) as it has been shown to be the most appropriate formula in dogs (57), provides the most consistent correction across a wide range of HR (58), and is simple in comparison with others. Our study demonstrated a significant increase in the QTc interval in both propofol and alfaxalone groups following induction of anesthesia, with no significant difference between the drugs. However, with a reported reference interval between 150 to 240 ms in dogs (23), ~60% of the dogs (33 out of 54) showed an increased QTc interval following premedication but before induction. This finding might suggest either a pre-existing abnormality in these animals, or an effect of the agents used for premedication. Given the high incidence of this phenomenon, it is very unlikely to be related to underlying pathology, and more probably drug-induced. Methadone has previously been shown to increase the QT interval in humans (59), and, more recently, in dogs (28). Methadone blocks cardiac potassium channels and subsequently interrupts the delayed rectifier potassium current, which prolongs the action potential and delays ventricular repolarization, manifesting as a prolonged QT interval on the ECG (60). It is also possible that acepromazine may have played a role. An experimental study in humans reported that acepromazine may have a potentially arrhythmogenic action through the inhibition of human ether-à-go-go-related gene (hERG) potassium channels in cardiac tissues and the prolongation of QT intervals (61). We elected to use methadone as premedication in the dogs in this study as it is the only full opioid agonist currently licensed in this species in the U.K. and Europe, and we wanted to produce data relevant to clinical veterinary practice. The observed prolongation of the QTc interval in both groups can be traced back to the extension of its constituent components: the QRS and JTc intervals, representative of ventricular depolarization and repolarization, respectively. Induction to general anesthesia led to the lengthening of both intervals across both groups. A proposed cellular mechanism for this QT prolongation implicates the inhibition of delayed rectifier potassium currents, causing a diminished potassium efflux from cardiomyocytes and subsequently increasing the ventricular action potential (AP) duration (62). A causative role in QTc prolongation with propofol administration has been demonstrated *in vivo* and *in vitro* human studies (63, 64). Propofol has been reported to affect the activity of several ion channels. Some of these interactions are thought to contribute to its anti-arrhythmic and cardio-protective properties, particularly evident in mitigating damage during myocardial ischemia (65–67). Experimental investigations on human (68), rat (69), dog (70) and guinea pig (71–73) cardiomyocytes have demonstrated that propofol reduces calcium Ca^2+^ influx by inhibiting L-type voltage-dependent Ca^2+^ channels. However, this reduction results in the shortening of the phase 2 of the cardiac AP, leading to a decrease in the AP duration (71–73). Inhibition of the slowly and rapidly activating delayed rectifier K^+^ current (I_Kr_, I_Ks_) has also been confirmed in both animal and human experimental studies (73–75). This phenomenon results in the prolongation of the phase 2 and 3 of the cardiac AP, thereby increasing the AP duration. Notably, genetic mutations affecting these ion channels represent the most common cause of Long QT Syndrome (LQTS) in humans (73, 76). Moreover, propofol demonstrates an inhibitory effect on the cardiac Na^+^ channel Na_v_1.5 (54), a membrane protein responsible for the I_Na_ current triggering cardiac cell depolarization. Mutations in this channel have been identified as causal factors for a variant of LQTS in humans (76). This intricate network of interactions sheds light on the multifaceted impact of propofol on cardiac electrophysiology. Despite the vast body of literature on interaction between propofol and ion channels, to the authors' knowledge, there are no electrophysiology studies in either the human or the veterinary literature describing relationships between alfaxalone and ion channels, or any other mechanisms that could interfere with the ventricular action potentials. Similarly, there are limited clinical data regarding the ECG effects of alfaxalone in human medicine (77). While our study reports for the first time a prolongation of the QTc in dogs following alfaxalone, the precise mechanism for this effect remains unclear, and further studies are also required to establish if a similar situation occurs in humans.

Alfaxalone administration caused a statistically significant ST segment depression; however, this resulted in a value out with the reference (±0.2 mV) in only one dog (−0.27 mV). In veterinary medicine this parameter has been given very little attention because of the low incidence of myocardial infarction and coronary artery disease in domestic species (78). However, during anesthetic procedures, changes in the ST segment can have important clinical implications, possibly indicating impaired myocardial oxygenation (79). Alfaxalone (in combination with alphadolone) has been reported to cause ST segment depression in humans (19), but the underlying mechanism has not been elucidated. It is possible that the significant increase in HR observed with alfaxalone might increase myocardial oxygen consumption, causing ST segment depression, although the clinical relevance is unknown.

The T waves analyzed in this study follow the qualitative and quantitative features of the normal canine population T waves (80). The T wave is one the most variable waves in the electrocardiogram and may change under several pathological conditions (81) or in association with altered autonomic tone, with variable and opposing effects reported (82–84). A reduction in T-wave amplitude has been observed after thiopentone administration in humans (85) and dogs (14). Initial data analysis from this current study showed a significant increase in the T wave amplitude in the alfaxalone group. However, due to the variation in polarity of the T wave from animal to animal, the T waves were subsequently grouped into positive and negative polarities and then re-analyzed, which revealed no significant differences in T wave amplitude either within or between the groups.

Different leads of the standard 12-lead ECG project the repolarization signals of different regions of the myocardial tissue (86). The QTd has been proposed as a measure of the range of this interlead variability (87). Indeed, it is hypothesized that the QTd reflects regional differences of ventricular repolarization (88). Intrinsic differences in the action potential duration of endocardial, mid-myocardial (M cells) and epicardial cells contribute to a physiological phenomenon known as ventricular transmural dispersion of repolarization (TDR). This heterogeneity of action potentials across different layers of the ventricular myocardium is due to a variable expression of ion channels and explains the reason why the repolarization process occurs unevenly in the heart (89). Even in the absence of abnormal anatomical substrates, cardiomyocytes exist in different states of excitability and refractoriness across the ventricular wall, generating different excitable myocardial areas, resulting in an increased TDR. This translates to an exaggerated heterogeneity and predisposes to arrhythmias because of trigger activity (via early afterdepolarization) and functional re-entry (90). Re-entrant circuits allow the generation of abnormal impulse conduction and are believed to be the main mechanisms of ventricular tachyarrhythmias and sudden cardiac death (91, 92). No significant difference in QTd was found either within groups (pre- to post-induction), or between groups, suggesting that both propofol and alfaxalone might have minimal impact on the inhomogeneity of ventricular repolarization and refractoriness. Lack of impact of propofol on QTd has previously been reported in dogs (14), although the values documented in our study are slightly higher compared to those reported by Dennis et al. (14) (17.21 ms pre-induction, 16.99 ms post-induction). However, is difficult to fully interpret these differences as there is no reported reference interval currently available for QTd in dogs.

Our study has some potential limitations. Ideally, when evaluating the effects of an anesthetic induction agent on the ECG, no premedication should be administered, so it is solely the impact of the hypnotic agent, itself, being assessed. Drugs used for premedication probably exerted some influence on the ECG parameters, which might have affected the results obtained. However, the absence of premedication would have resulted in an increased dose requirement for the induction agent, with consequent greater cardiorespiratory depression and is unacceptable in client-owned animals. Given that the premedication protocol was identical for all the dogs, any changes observed between the ECG values obtained pre-induction (post-premedication) and post-induction, should, theoretically, be in response to the hypnotic agent itself, therefore minimizing bias between the groups. Measurement bias is an important limitation of this study as the method used can strongly influence the QT interval (93). Identifying the intersection between the descending branch of the T wave and the isoelectric line can be challenging for trained eyes and also computer algorithms (22, 94). Despite the fact that all measurements were performed by the same operator after a period of training under direct supervision of an EBVS Specialist in Veterinary Cardiology, we cannot completely rule out a degree of intra-individual variability in measurement technique. We also cannot completely exclude the presence of pre-existing structural/functional heart disease or transient arrhythmias in the dogs included in the study, as their absence was based solely on clinical examination and no Holter monitoring or echocardiographic examination was performed. Lastly, the lack of significant difference in QTd in both groups may simply reflect the inability of this parameter to correlate to the electrical and spatial inhomogeneity of the myocardium. More studies are needed to better characterize this parameter in veterinary medicine.

In conclusion, this study demonstrates, for the first time, that alfaxalone, similar to propofol, causes QTc prolongation in dogs, and may, therefore, exhibit arrhythmogenic properties. No significant difference in QTd could be demonstrated with either drug, suggesting that both propofol and alfaxalone might have minimal impact on the inhomogeneity of ventricular repolarization, although more research in this area is required.

## Data availability statement

The raw data supporting the conclusions of this article will be made available by the authors, without undue reservation.

## Ethics statement

The animal studies were approved by RCVS Ethics Review Panel (Ref: 2021-34). The studies were conducted in accordance with the local legislation and institutional requirements. Written informed consent was obtained from the owners for the participation of their animals in this study.

## Author contributions

VC: Conceptualization, Data curation, Formal analysis, Methodology, Project administration, Visualization, Writing—original draft, Resources, Supervision. VG: Conceptualization, Writing—review & editing, Methodology. AA: Conceptualization, Formal analysis, Funding acquisition, Methodology, Writing—review & editing. SM: Data curation, Resources, Writing—review & editing. DF: Conceptualization, Methodology, Writing—review & editing.
